# Characterization of amiodarone action on currents in hERG-T618 gain-of-function mutations

**DOI:** 10.1515/biol-2022-0749

**Published:** 2023-11-03

**Authors:** Min Lin, Cuiyun Li, Chao Lin, Shangquan Xiong, Qiao Xue, Yang Li

**Affiliations:** Department of Cardiology, People’s Hospital Affiliated to Fujian University of Traditional Chinese Medicine, Fuzhou, 350004, China; Department of Cardiology, Chinese PLA General Hospital, No. 28 Fuxing Road, Haidian District, Beijing, 100853, China

**Keywords:** amiodarone, HEK293 cells, hERG-T618I current, patch clamp technique

## Abstract

The purpose of this study was to determine the effect of amiodarone (Ami) on hERG-T618I currents in HEK293 cells. A transient transfection method was used to transfer hERG-T618I and hERG wild-type (WT) channel plasmids into HEK293 cells. An extracellular local perfusion method was used for administration. Currents were recorded using the whole-cell patch clamp technique. Ami (10 μM) had a greater retarding effect on the hERG-T618I channel than WT (*P* < 0.05). The half-inhibitory concentration for the mutant was approximately 1.82 times that of WT (*P* < 0.05). The WT current inhibition fraction against Ami was significantly greater than T618I in the same cell (*P* < 0.05). The STEP current of the mutant channel was larger than the WT channel, but the characteristic of inward rectification did not appear. Ami reduced the STEP current of the mutant channel, and the steady-state activation curve indicated that channel activation decreased (*P* > 0.05). Ami restored partial inactivation of the mutant channel. Ami effectively reduced the current in the phase 2 plateau (*P* < 0.05), but the phase 3 current did not exhibit the characteristics of a WT current. Ami inhibited hERG-T618I currents on HEK293 cells, but the effect was weaker than WT.

## Graphical abstract

Ami (10 μM) had a greater retarding effect on the hERG-T618I channel than WT (*P* < 0.05). The half-inhibitory concentration for the mutant (4.56 μM) was approximately 1.82 times that of WT (2.51 μM).

**Figure:**
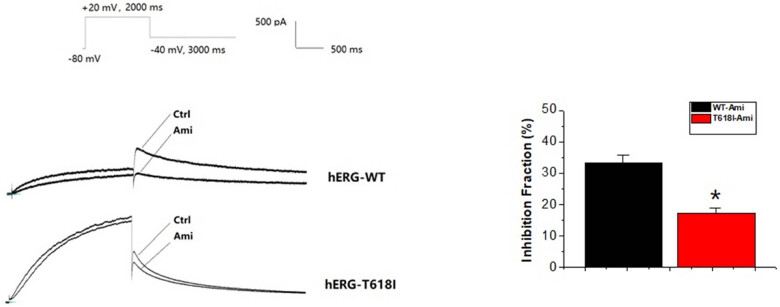

## 1 Introduction

The human ether-à-go-go-related gene (hERG) encodes the pore-forming α-subunit of the rapidly activating delayed rectifier potassium channel (*I*_Kr_) in cardiomyocytes [1,2]. The KCNE2 gene encodes the β-subunit (MiRP1) of the *I*_Kr_ channel [1,2]. The two subunits assemble to form wild-type (WT) *I*_Kr_, which conducts the major current responsible for action potential (AP) repolarization in cardiomyocytes and contributes to phase 3 of the repolarization process [1,2]. The magnitude of the *I*_Kr_ current determines the cardiomyocyte AP duration. The larger the current, the shorter the cardiomyocyte AP duration; otherwise, the AP duration is prolonged [3]. Therefore, the hERG-T618I gain-of-function mutation results in an increased density of the *I*_Kr_ current and the short QT syndrome [SQTS] [4]. The *I*_Kr_ current is the major current inducing electrical remodeling in several heart diseases and is closely related to malignant arrhythmias and sudden cardiac death [5]. The *I*_Kr_ current is also the primary target for class III antiarrhythmic drugs [5]; however, the SQTS caused by hERG mutations is refractory to class III antiarrhythmic drugs, such as sotalol and ibutilide, but moderately sensitive to class Ia antiarrhythmics, such as quinidine and disopyramide [6].

Amiodarone (Ami) is a potent drug that has been extensively used against fast arrhythmias in the clinical setting for nearly 30 years. With high efficacy and a low pro-arrhythmogenic effect, Ami occupies an important position among antiarrhythmic drugs and has been recommended as first-line therapy in many guidelines, despite the associated toxicity [7,8]. Ami is a class Ⅲ antiarrhythmic that blocks multiple potassium channels, including the *I*_Kr_, the slowly activating delayed rectifier potassium current (*I*_Ks_), and the transient outward potassium current [*I*_to_] [9,10]. The hERG-T618 gain-of-function mutation is associated with a distinctly impaired sensitivity to several antiarrhythmics compared to WT hERG [11]. Thus, the hERG-T618 gain-of-function mutation has a direct impact on the efficacy of antiarrhythmics [11]; however, sensitivity to Ami in the presence of a hERG-T618 gain-of-function mutation has not been reported. We compared sensitivity to Ami with and without a hERG-T618I gain-of-function mutation, thus providing experimental clues to promote rational drug use.

## 2 Materials and methods

### 2.1 Drugs and reagents

HEPES, pronase E, l-glutamic acid, K-aspartate, GTP, CdCl_2_, TTX, CsCl, and dofetilide were purchased from Sigma-Aldrich (St. Louis, MO, USA). EGTA and DMEM were purchased from Solarbio (Beijing, China). Fetal bovine serum, lipofectamine transcription reagent, and green fluorescent protein (GFP) were purchased from Invitrogen (Carlsbad, CA, USA).

Ami (MW = 681.77) was purchased from Sigma-Aldrich. Ami was dissolved in dimethyl sulfoxide and made into a stock solution, which was later diluted using extracellular solution to 6 different concentrations (0.1, 0.3, 1.0, 3.0, 10.0, and 30.0 μM). The Ami solution was administered in the extracellular constant current perfusion mode using a local perfusion device. To detect the effect of DMSO on current, *I*_hERG_ current was recorded when extracellular solution contained 0 (total solution volume: 3,000 μL) and 25.0 μL (total solution volume: 3,025 μL, 0.82% v/v), respectively, and it was not found that there was a significant change in current amplitude under the two conditions (*n* = 5, *P* > 0.05). In this experiment, local perfusion model of cells was used with a local perfusion device. To achieve the equilibrium of drug concentration in extracellular solution and the steady effect of Ami on current, *I*_hERG_ current was recorded after 5 min of drug perfusion at room temperature (23–25°C).

The amplitude of the tail current before and after drug application was recorded in the same cell, and the current density was calculated using the cell capacitance. The percent inhibition of the peak tail current by Ami was measured. Inhibition fraction of drug (%) = (*I*_before_ − *I*_after_)/*I*_before_ × %; *I*_before_ is the amplitude of the tail current before drug application and *I*_after_ is the amplitude of the tail current after drug application. Then, the inhibition fraction (%) of the tail currents was compared between WT and T618I. We also determined tail-current activation kinetics and calculated the activation time constant (tau) using an exponential fitting (*I* = *I*∞[1 − exp (1 − *t*/tau)]. The voltage dependence of WT and T618I channel activation was compared.

### 2.2 Solution preparation

The pipette solution contained (mmol/L): K-aspartate, 140; MgATP, 4; MgCl_2_, 1; EGTA, 10; GTP, 0.1; and HEPES, 10. The pH was adjusted to 7.3 with KOH.

The extracellular solution contained (mmol/L): NaCl, 140; KCl, 4; CaCl_2_, 1; MgCl_2_, 1; HEPES, 10; and glucose, 5. The pH was adjusted to 7.4 with NaOH.

### 2.3 HEK293 cell culture and transfection with the pcDNA3.1-hERG plasmid

HEK293 cells (ATTC, Manassas, VA, USA) were cultured. Cell cultures with good growth status were observed under a light microscope and had the following features: triangular or rhombus shaped; multiple protrusions extending outwards; clear and transparent cytoplasm; and no intracellular or extracellular impurity particles. Transfection was conducted using the Lipofectamine™ 2000 Transfection Reagent. The target plasmids were pcDNA3.1-hERG-WT (WT) and pcDNA3.1-hERG-T618I. The positive plasmid was pcDNA3.1-GFP. HEK293 cells harvested 48–72 h post-transcription. The cells were washed once with phosphate-buffered saline and digested with pancreatin for approximately 1 min. The reaction was terminated by adding culture medium. The cells were passaged at a 1:10 ratio to a 35-mm Petri dish and cultured in an incubator for 5 h. The transfected cells were observed under a fluorescence microscope and subsequently used for whole-cell patch-clamp recording.

### 2.4 Current record using the whole-cell patch-clamp recording method

Time dependent characteristics of current: to avoid changes in *I*_Kr_ current caused by instability of membrane resistance and other conditions during recording period, *I*_Kr_ current was recorded at 0, 5, 15, and 30 min after membrane breaking. It was not found that currents of WT and T618I significantly change within this time range. All the subsequent experiments were completed within ∼30 min after membrane rupture (Figure S1).

To detect the effect of DMSO on current, *I*_Kr_ current was recorded when extracellular solution contained 0 (total solution volume: 3,000 μL) and 25.0 μL (total solution volume: 3,025 μL, 0.82% v/v), respectively, and it was not found that there was significant change in current amplitude under the two conditions (*n* = 5, *P* > 0.05, Figure S2).

AP was recorded in the current-clamp mode and the currents were recorded in the voltage-clamp mode. AP was stimulated to the epicardial myocardium of guinea pig, with an AP duration of approximately 180 ms. An Axon-700B clamp amplifier (Molecular Devices, Sunnyvale, CA, USA) was connected to the computer. The stimulation signals and voltage input signals were acquired using a Digidata 1440A Digitizer (Molecular Devices) and pCLAMP10.4 software. A GG-17 glass blank was drawn into a 2.0–5.5 MΩ electrode using a pp-83 glass microelectrode puller (Narishige Co., Tokyo, Japan). The 3D manipulator was adjusted for the membrane seal with a seal resistance >1.0 GΩ. Suction was applied to disrupt the membrane patch to establish the whole-cell mode. For capacitance measurement, a ramp stimulus of 0.4 V/s was applied. The current was measured and the membrane capacitance was calculated using the following equation: *C*_m_ = *I*/(d*V*/d*t*), where *C*_m_ is the membrane capacitance, *I* is the current, and d*V*/d*t* is the voltage slope. The value of *I* was represented by the current density (pA/pF) to eliminate errors between cells. The signals were passed through a fourth-order Bessel active low-pass filter with a cut-off frequency of 1 kHz and a sampling frequency of 5.0 kHz. Voltage deviation was eliminated by a series resistance compensation of 90–95%. The liquid junction potential was corrected so that the liquid junction potential was <2.0 mV. The influence of charging and discharging the membrane capacitance was eliminated by a slow capacitance compensation of 85–90%.

### 2.5 hERG current recording procedure and data analysis

The potential was maintained at −80 mV, and a depolarizing pulse of +20 mV for 2,000 ms was applied. The *I*_hERG,step_ current was recorded. The time was 3,000 ms when the pulse was repolarized to −40 mV. The *I*_hERG,tail_ current was recorded. This current was completely blocked by 1 μM dofetilide.

The potential was maintained at −80 mV, and a depolarizing pulse of −50 to +50 mV for 2,000 ms was applied with increments of 10 mV. The time was 3,000 ms when the pulse was repolarized to −40 mV. The *I*_hERG,tail_ current was recorded and the current density was calculated. The stimulation pulse at each voltage was plotted on the *x*-axis, and the current density on the *y*-axis. The current–voltage (*I*–*V*) dependence was observed.

The potential was maintained at −80 mV. A depolarizing pulse of +40 mV for 300 ms was applied, which was followed by a short-term hyperpolarization stimulus of −120 mV for 10 ms. This stimulus was further followed by a test stimulus of −70 to +20 mV for 100 ms. The *I*_hERG,tail_ was recorded, and the inward rectification of the current was observed.

### 2.6 Statistical analysis

All data are expressed as the mean ± SD and processed using pCLAMP10.4 software. The data were statistically analyzed using SPSS21.0 software. Analysis of variance was used for multiple comparisons, and a Student–Newman–Keuls (SNK-q) test was performed for pairwise comparisons. A *P*-value of <0.05 was statistically significant.

The inhibition fraction of the peak tail current was measured. The graph is draw with the inhibitory fraction against the logarithm of the drug concentration and the mean data points were fitted with the Hill equation. The concentration of drugs needed to yield a 50% blockade of the hERG current (IC_50_) was obtained by fitting the data to a Hill equation: *I*/*I*_0_ = 1/[1 + ([*C*]/IC_50_)^*n*H^], where *I*_0_ and *I* are the current amplitudes measured in the absence and presence of drugs, respectively, [*C*] is the concentration of drugs in the external solution, and *n*H is the Hill coefficient.

## 3 Results

### 3.1 Action of Ami on the hERG-T618I current density

**Figure 1:**
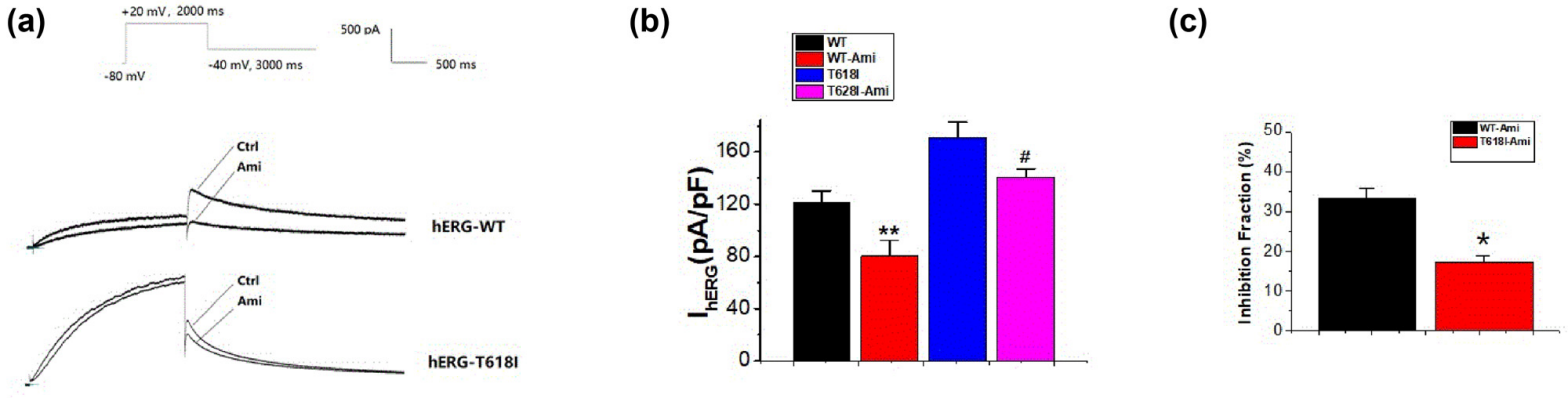
Action of Ami on the hERG-T618I current density. (a) Representative hERG-T618I and hERG-WT current traces recorded from cells under control conditions and after the addition of 10 μM Ami; (b) after the addition of 10 μM of amiodarone (Ami), the current density of *I*_hERG-WT,tail_ and *I*_hERG-T618I,tail_ decreased significantly (all *P* < 0.05). The current density for the *I*_hERG-WT,tail_ decreased from 120.74 ± 9.27 to 80.61 ± 12.51 pA/pF (*P* < 0.01). The current density for the *I*_hERG-T618I,tail_ decreased from 171.17 ± 11.55 to 140.90 ± 6.17 pA/pF (*P* < 0.05); (c) the inhibition fraction (%) of the tail currents was compared between WT and T618I (*P* < 0.05).

The *I*_hERG-WT,tail_ and *I*_hERG-T618I,tail_ decreased after the addition of 10 μM Ami (Figure 1a). At +50 mV, the current density for the *I*_hERG-WT,tail_ decreased from 120.74 ± 9.27 to 80.61 ± 12.51 pA/pF (an approximate proportion of 33.24% ± 2.31% [*n* = 15]; *P* < 0.01). The current density for the *I*_hERG-T618I,tail_ decreased from 171.17 ± 11.55 to 140.90 ± 6.17 pA/pF (an approximate proportion of 17.29% ± 1.78% [*n* = 15]; *P* < 0.05) (Figure 1b). The amplitude of the tail current before and after drug application was recorded in the same cell, and the current density was calculated using the cell capacitance. The percent inhibition of the peak tail current by Ami was measured. Inhibition fraction of drug (%) = (*I*_before_ − *I*_after_)/*I*_before_ × %; *I*_before_ is the amplitude of the tail current before drug application and *I*_after_ is the amplitude of the tail current after drug application. Then, the inhibition fraction (%) of the tail currents was compared between WT and T618I (Figure 1c). This result indicated a significant difference in the Ami-induced blocking effect between WT and mutant currents.

### 3.2 Concentration-dependent inhibition of Ami on the hERG-T618I current

**Figure 2:**
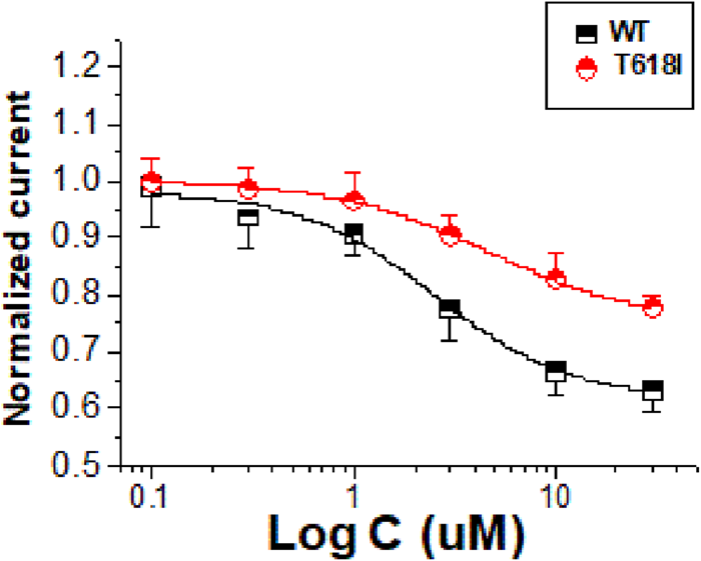
A concentration-dependent action of the effects of Ami (0.1, 0.3, 1.0, 3.0, 10.0, and 30.0 μM) on the hERG-T618I and hERG-WT current. The IC_50_ for the *I*_hERG-WT,tail_ was 2.51 μM and a Hill coefficient of 1.15 (*n* = 12). The IC_50_ for the *I*_hERG-T618I,tail_ was 4.56 μM and the Hill coefficient was 1.30 (*n* = 12). The IC_50_ for the mutant current was approximately 1.82 times the WT current (*P* < 0.05).

Ami was added to the extracellular solution at 6 different concentrations (0.1, 0.3, 1.0, 3.0, 10.0, and 30.0 μM), and then, the effect on currents was observed. The concentration-dependent action of Ami on the *I*_hERG-WT,tail_ was observed, with a half maximal inhibitory concentration (IC_50_) of 2.51 μM and a Hill coefficient of 1.15 (*n* = 12). The IC_50_ for the *I*_hERG-T618I,tail_ was 4.56 μM and the Hill coefficient was 1.30 (*n* = 12). The IC_50_ for the mutant current was approximately 1.82 times the WT current (*P* < 0.05; Figure 2).

### 3.3 Time-dependent characteristics of Ami inhibition

**Figure 3:**
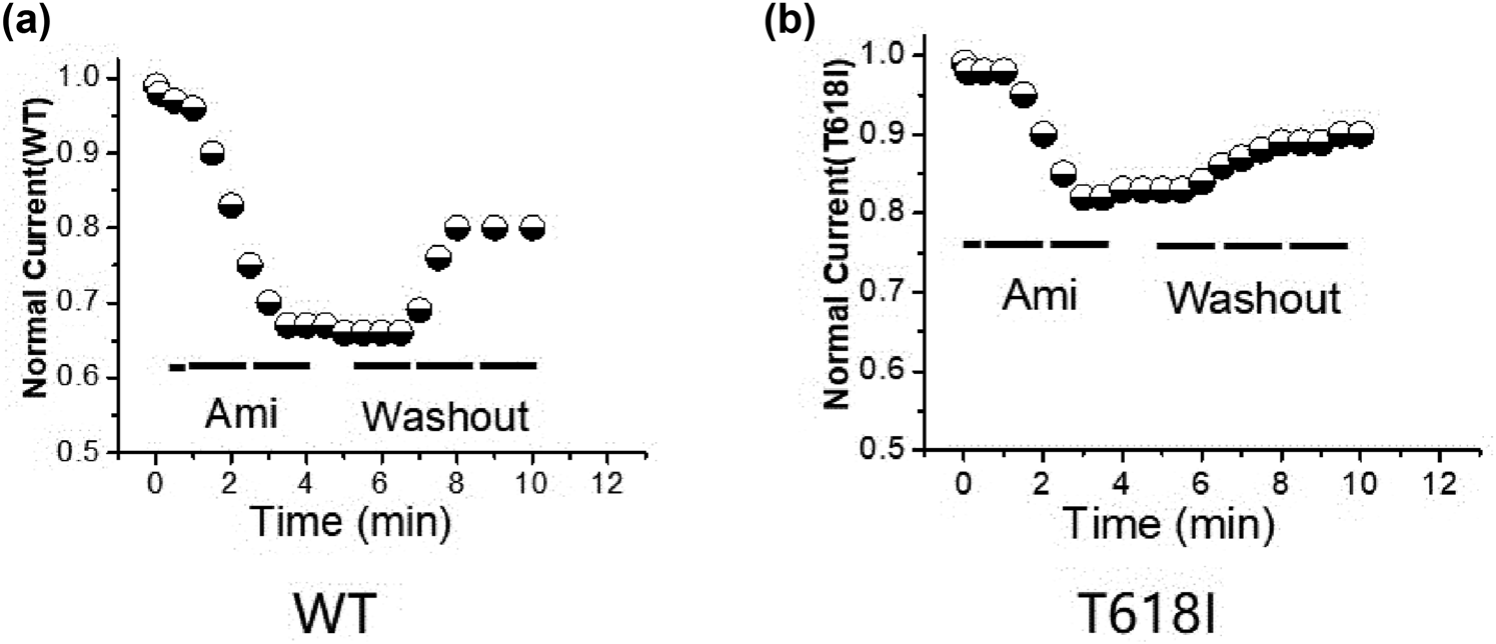
Time-dependent characteristics of Ami inhibition. (a) After 10 µM Ami was applied to perfuse the bath, the maximal inhibition was 33.2% of the control current. With the drug-free solution washout, the amplitude of hERG-WT current recovered to 80.0% of the control level; (b) after 10 µM Ami was applied to perfuse the bath, the maximal inhibition was 17.2% of the hERG-T618I current. With the drug-free solution washout, the amplitude of hERG-T618I current was reversed with 90% recovery within 10 min.

The experiments were performed to identify time-dependent features of hERG-WT and hERG-T618I mutation inhibition and washout action of the Ami. After a control period of 2 min, which demonstrated the stability of the experimental conditions, 10 µM Ami was applied to perfuse the bath. The maximal inhibition with 33.2% of the control current occurred within 4 min, and a steady-state block was obtained with a small further increase in inhibition. With the drug-free solution washout, the amplitude of hERG-WT current recovered to 80.0% of the control level in approximately 10 min. In another experiment, after a control period of 2 min, 10 µM Ami was added to the bath solution of hERG-T618I mutation cells. The maximal inhibition with 17.2% of the control current occurred within 4 min, and a steady-state block was obtained. The drug was washed out, and the current was reversed with 90% recovery within 10 min (Figure 3).

### 3.4 Effect of Ami on activation of hERG-I618I mutant channels

**Figure 4:**
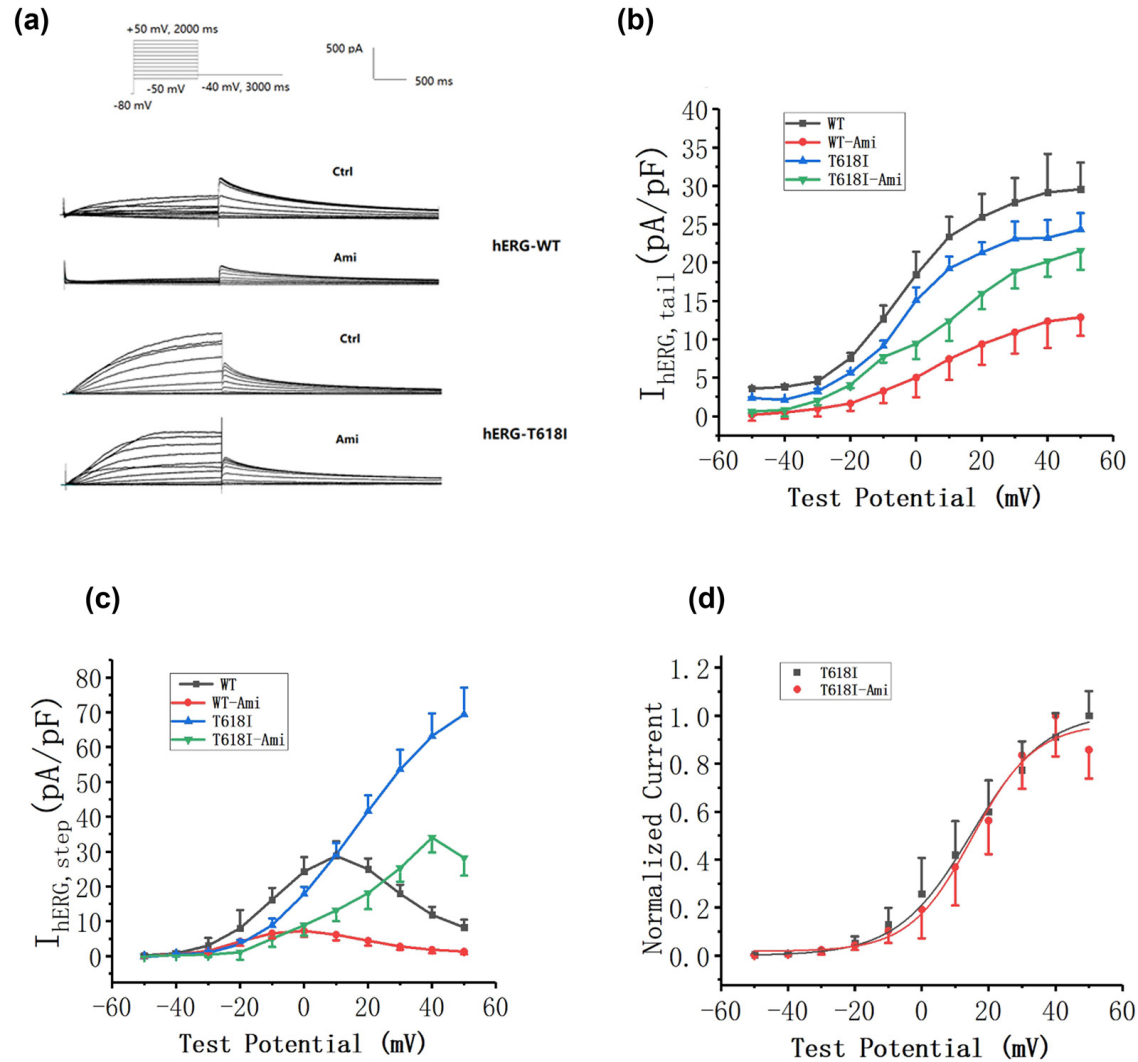
Effect of Ami on activation of hERG-I618I mutant channels. (a) Representative hERG-T618I and hERG-WT current traces recorded from cells under control conditions and after the addition of 10 μM Ami; (b) after the addition of 10 μM Ami, the decrease in the tail current density was more significant for the *I*_hERG-WT,tail_ than the *I*_hERG-T618I,tail_, which was even more pronounced at >+20 mV (*P* < 0.05); (c) after the application of amiodarone, the inward rectification characteristics did not change in the WT channel in the STEP current stage but did not show current attenuation; however, the voltage amplitude of the channel decreased significantly (*P* < 0.05). (d) STEP current showed a steady-state activation curve sloping to the right, which was different from the WT channel after drug application (*P* > 0.05).

As the stimulation pulse voltage shifted towards the positive potential, the current densities increased for the *I*_hERG-WT,tail_ and *I*_hERG-T618I,tail_. Stability was reached when the stimulation potential was +20 mV. Both currents decreased after the addition of 10 μM Ami. The decrease in the tail current density was more significant for the *I*_hERG-WT,tail_ than for the *I*_hERG-T618I,tail_ (Figure 4a and b; *n* = 15, *P* < 0.05). The WT channel showed inward rectification in the STEP current stage, and the application of Ami only changed the current amplitude, but the inward rectification characteristics did not change. This result suggests that Ami acted on the activation of the channel. The WT channel did not show current attenuation at the STEP current stage, indicating that the channel remained open without inactivation. The voltage amplitude of the channel decreased significantly after the application of Ami, indicating that Ami significantly inhibited activation of the mutant channel (Figure 4c; *n* = 15, *P* < 0.05). Inactivation of the mutant channel disappeared, so the STEP current was used directly to make a steady-state activation curve. Therefore, a steady-state activation curve sloping to the right for the STEP current, which was different from the WT channel, as follows: T618I *V*_1/2_ = 14.86241 ± 1.76483 (*n* = 9), curve slope *k* = 11.10278 ± 0.62824 (*n* = 9); T618I with Ami *V*_1/2_ = 15.07437 ± 2.19289 (*n* = 9), curve slope *k* = 9.34893 ± 1.95032 (*n* = 9, Figure 4d; *P* > 0.05).

### 3.5 Effect of Ami on inactivation of hERG-I618I mutant channels

**Figure 5:**
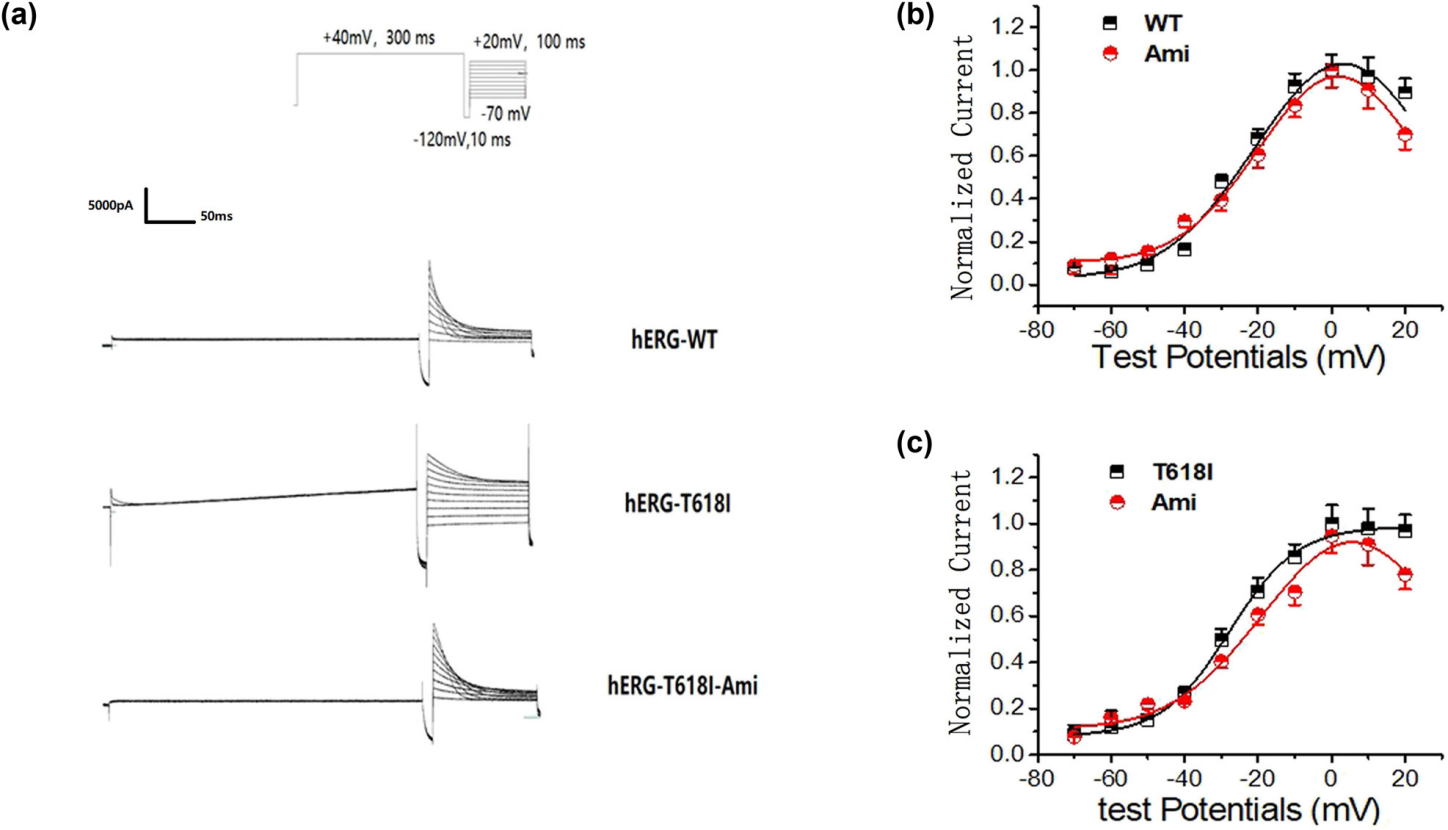
Effect of Ami on inactivation of hERG-I618I mutant channels. (a) Representative hERG-I618I and hERG-WT current traces recorded from cells under control conditions and after the addition of 10 μM Ami; (b) after treatment, the WT standardized current decreased from 0.85 ± 0.07 to 0.74 ± 0.07 (approximately 11.25%) at +20 mV; (c) after treatment, the normalized current of the mutant type decreased from 0.97 ± 0.07 to 0.78 ± 0.07 (approximately 19.59%) at +20 mV. Ami had a greater effect on the inward rectification of the mutant type than the WT (Figure 5b and c; *n* = 10; *P* < 0.05).

When the current retest voltage of T618I was at 0 mV, the voltage value was the maximum. Therefore, for convenience, 0 mV voltage was also selected for voltage standardization in the WT channel. The *I*_hERG-T618,step_ gradually decreased when the stimulation tended to be 0 mV due to inward rectification. As the depolarizing voltage further increased, the *I*_hERG-T618,step_ decreased significantly. No considerable influence was observed on rectification after the use of Ami; however, we showed that the *I*_hERG-T618I,step_ current exhibited minimal inward rectification. As the depolarizing voltage increased, the current density increased. The inward rectification of the current was partially restored after the use of Ami (Figure 5a; *n* = 10).

The WT standardized current of 0.85 ± 0.07 was lower than the mutation current (0.97 ± 0.07) at +20 mV, suggesting that the mutation current was significantly reduced due to inward rectification. This finding may be the main reason for the hERG STEP current increase. After treatment, the WT standardized current decreased to 0.74 ± 0.07 (approximately 11.25%). The normalized current of the mutant type decreased to 0.78 ± 0.07 (approximately 19.59%). Ami had a greater effect on the inward rectification of the mutant type than the WT (Figure 5b and c; *n* = 10; *P* < 0.05).

The fast inactivation of hERG channels was represented as a characteristic of inward rectification in the STEP current phase. The partial recovery of inward rectification of the mutant channels by Ami suggested that Ami partially restored inactivation of the channel, but this effect was incomplete.

### 3.6 Action of Ami on the hERG-T618I current using the AP clamp

**Figure 6:**
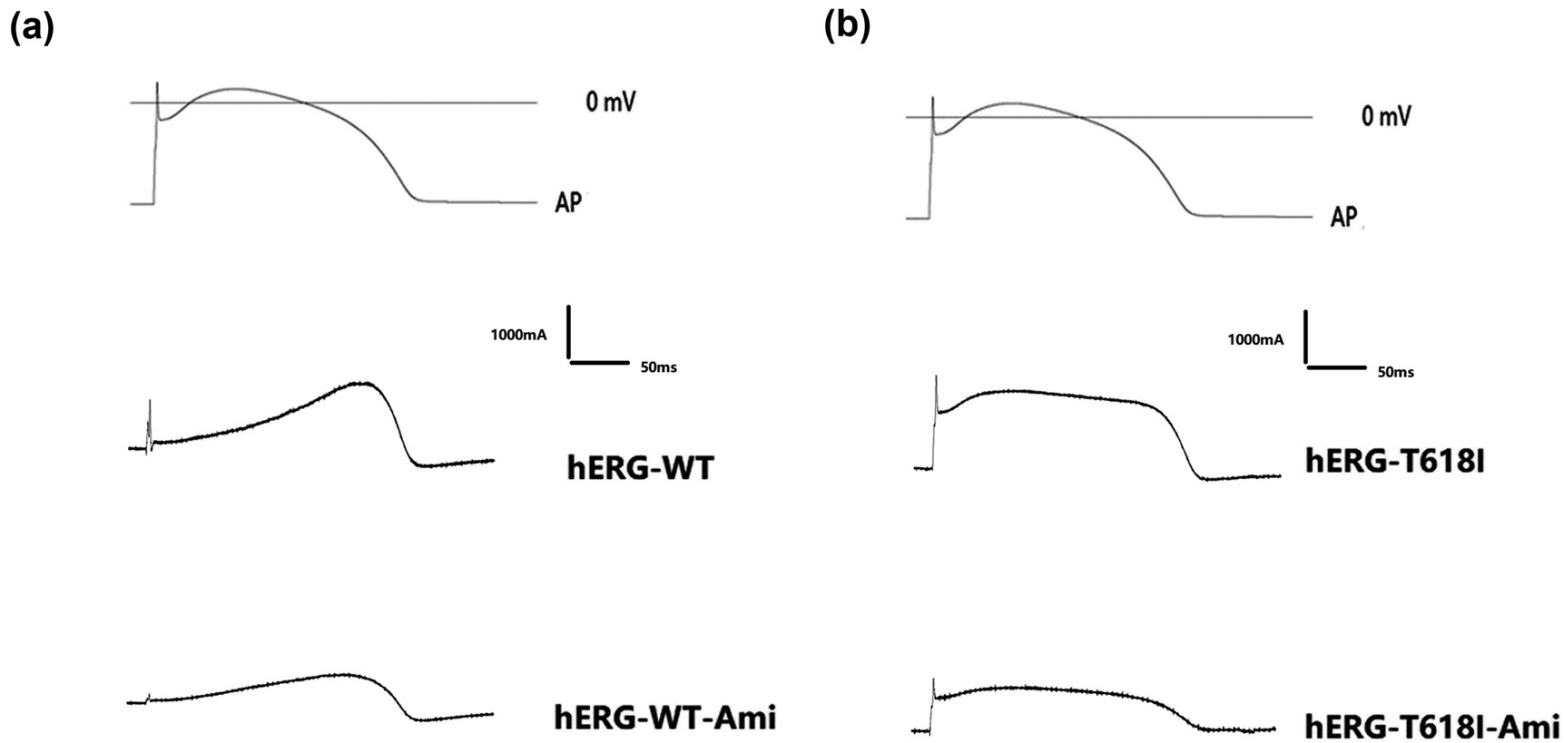
Action of Ami on the hERG-T618I and hERG-WT currents using the AP clamp. (a) Representative hERG-WT current traces recorded from cells under control conditions and after the addition of 10 μM Ami using the AP clamp and (b) representative hERG-T618I current traces recorded from cells under control conditions and after the addition of 10 μM Ami using the AP clamp. Both currents decreased after the use of Ami, and the decrease was greater for the T618I current in early repolarization.

Figure 6 shows the changes in WT and T618I currents when a ventricular AP was applied. In early repolarization, the WT current increased slowly; however, the increase was greater and reached a maximum in late repolarization (phase 3) (Figure 6a). The conductance of the T618I current increased rapidly in early repolarization and reached a high-level plateau that was maintained as the membrane potential was repolarized, followed by a gradual decline (Figure 6b). Both currents decreased after the use of Ami, and the decrease was greater for the T618I current in early repolarization.

**Figure 7:**
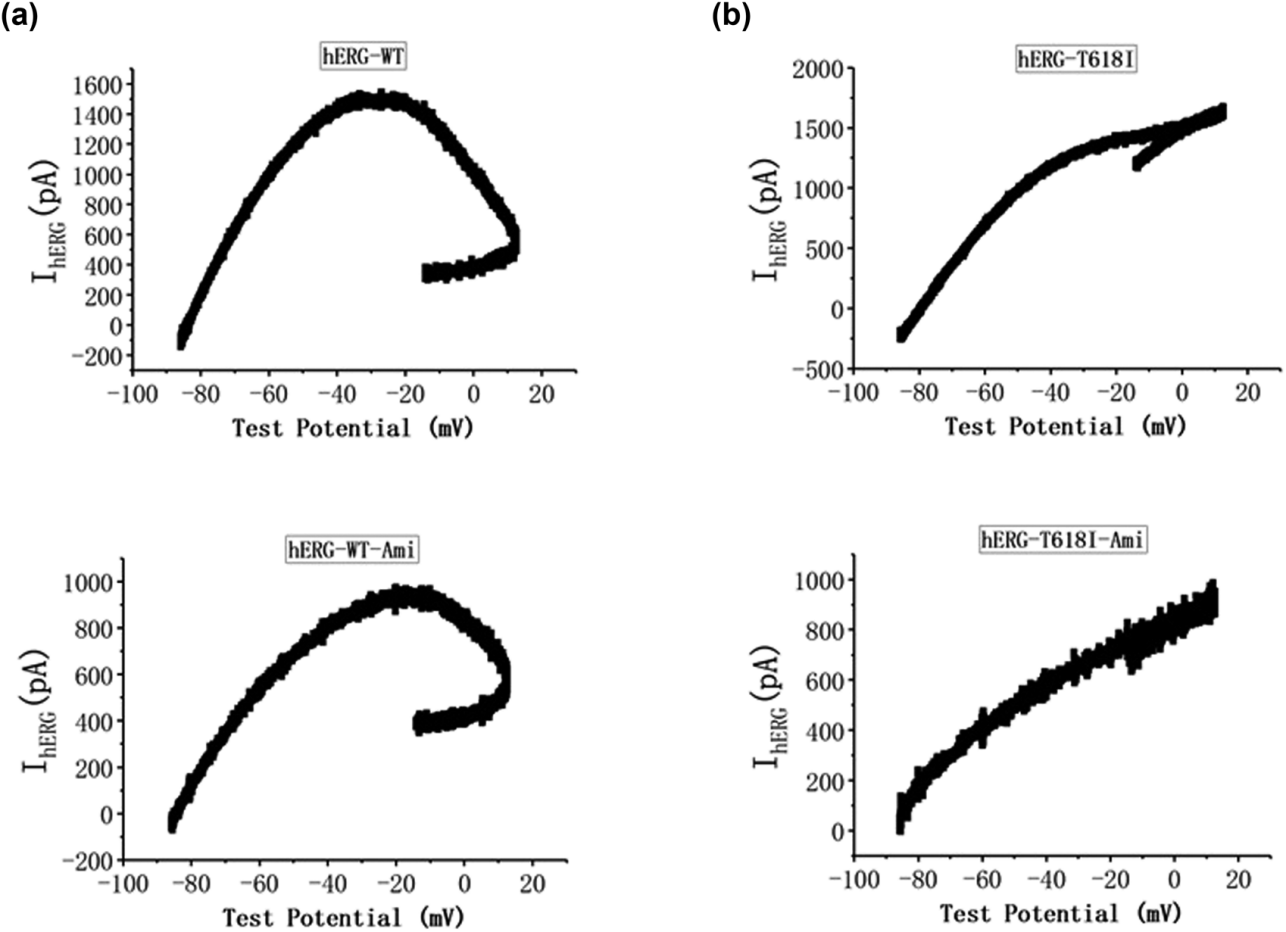
The voltage-dependent changes in hERG-WT and hERG-T618I currents when the ventricular AP clamp technique was used. (a) Representative current–voltage relationship for hERG-WT current under control conditions and after the addition of 10 μM Ami and (b) representative current–voltage relationship for hERG-T618I current under control conditions and after the addition of 10 μM Ami. After the application of amiodarone, the current peak decreased, but the form did not change, and the current distribution was similar to the WT channel.

Figure 7 shows the voltage-dependent changes in hERG-WT and hERG-T618I currents when the ventricular AP clamp technique was used. The hERG-WT current changed greatly in the range of −20 to +10 mV, which indicated that the two-phase plateau current of the AP rose slowly until the AP decreased to approximately −20 mV and reached the maximum current (Figure 7a). After Ami was given, the mutant current varied greatly in the range of −10 to +10 mV and reached the maximum when the AP reached +10 mV, indicating that the current peaked at the early stage of the two-phase platform of the AP (Figure 7b). After the application of Ami, the current peak decreased, but the form did not change and the current distribution similar to the WT channel also appeared. Specifically, the current peaked at the three-phase repolarization.

## 4 Discussion

SQTS is a rare hereditary disease that features an abnormally shortened QT interval in individuals with a normal heart structure. The most frequent presenting symptom of SQTS is cardiac arrest [12]. SQTS is the primary cause of atrial and ventricular arrhythmias and sudden cardiac death [12,13,14]. El-Battrawy et al. [14] reported that 71% of families with SQTS had a documented history of sudden cardiac death in a long-term follow-up study. In recent years, there have been numerous studies on the molecular mechanism underlying SQTS, which have provided evidence for diagnosing and treating SQTS [5,15–17]. According to the study by Du et al., the heterogeneity of ventricular-PF repolarization may be caused by the T618I mutation, which exacerbated differences in repolarizing IhERG between PF and ventricular Aps, and consequently resulted in the U waves seen in T618I carriers [5]. Abnormal shortening of the QT interval is caused by defective functioning of both potassium and calcium ion channels, of which potassium channels are most often affected [15,16]. An hERG-T618 gain-of-function mutation that occurs in hereditary or familial SQTS has a mild impact on repolarization of the cardiac AP [6]. According to El Harchi et al., the sensitivity of the hERG-T618I current to drug application decreased by varying degrees compared to the hERH-WT current [6]. For example, the IC_50_ of quinidine was 0.64 nM for hERG-WT and 0.88 nM for the hERG-T618I current. The IC_50_ of disopyramide was 7.68 nM for WT and 16.83 nM for T618I. A similar variation in the action on the hERH-WT and hERG-T618I currents was observed for D-sotalol and flecainide. The IC_50_ of sotalol for T618I was 3.2 times the IC_50_ for WT. The IC_50_ of flecainide for T618I was 2.5 times the IC_50_ for WT. Apparently, the drug-induced inhibition on the T618I current was moderately weakened [6]. Ami is an effective antiarrhythmic drug for treating atrial and ventricular arrhythmias. Although Ami is a class III antiarrhythmic drug, Ami affects nearly all phases of the cardiac AP [7,18]. Our study showed a reduced blocking effect of Ami on the hERG-T618I current. The inhibitory effect of Ami on the T618I current was approximately one-half of the action on the WT current at the same concentration (WT, 33.24 ± 2.31% vs T618I, 17.29 ± 1.78%). The IC_50_ for the mutant current was approximately 1.82 times the WT current. This result indicated that the dosage of Ami should be adjusted for SQTS caused by an hERG-T618I mutation to optimize efficacy.

The fast inactivation of the mutant channel disappeared, as shown by the continuous increase in the step current. Ami effectively reduced the step current amplitude. As indicated by the right shift of the steady-state activation curve, Ami made it more difficult to activate the channel and further reduced the activated current. In contrast, after the application of Ami, the mutant channel partially recovered the characteristics of inward rectification at a higher voltage, which also indicated a partial recovery of the inactivation of the channel, but this inactivation was incomplete. Both mechanisms are involved in the effect of Ami on mutant channels. Further AP clamp experiments confirmed that Ami significantly decreased the channel current in the early stage of AP repolarization but did not appear to slowly increase similar to the WT. This finding suggested that Ami mainly affected channel activation but did not have a major role in recovery of the channel inactivation function [15,16].

We only studied the action of Ami on the hERG-T618I current alone by excluding the interferences of all other currents. In this way, we more clearly understood how Ami acted on the hERG-T618I current; however, the electrical activity of the heart is the result of the coordinated action of several ion currents [19,20]. What may happen after the Ami-induced blocking of the hERG-T618I current remains to be further investigated.

To conclude, Ami inhibited the hERG-T618I current in HEK293 cells, but the inhibition was less intense than WT hERG. It is therefore necessary to adjust the dosage and treatment regimen if patients have SQT1 caused by a hERG-T618I mutation.

## Supplementary Material

Supplementary Figure
