# Supplementary material for: Characterization of amiodarone action on currents in hERG-T618 gain-of-function mutations
Source: Open Life Sci. 2023 Nov 3;18(1):20220749. doi: 10.1515/biol-2022-0749 (PMC10638844; doi:10.1515/biol-2022-0749)
Supplement: Supplementary Figure [file biol-2022-0749-sm.pdf]

Supplementary material

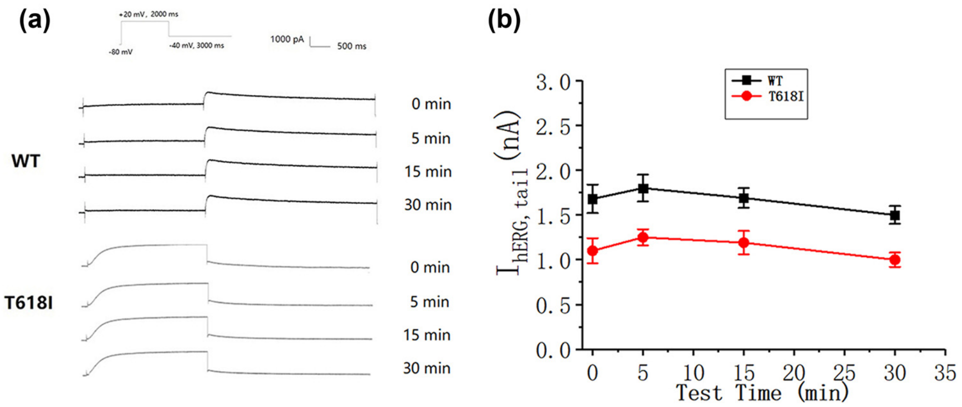

Figure S1: Time dependent characteristics of current. (a) Ikr currents of WT and T618I were recorded at 0, 5, 15 and 30 min after membrane breaking. (b) It was not found that the tail current density of WT and T618I significantly changes within this time range.

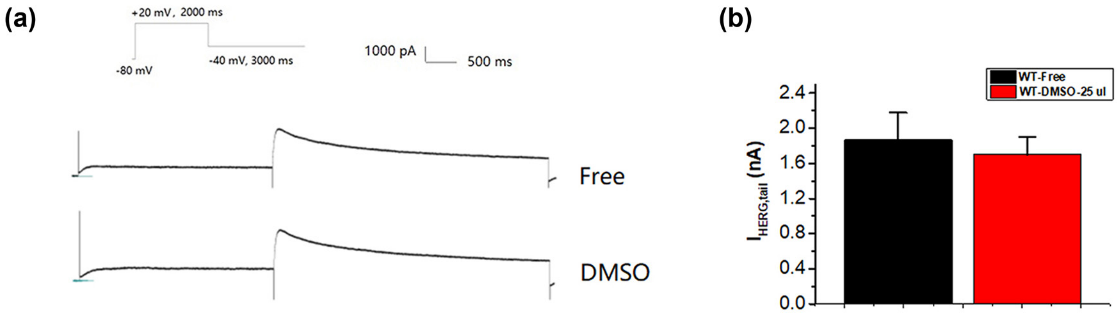

Figure S2: The effect of DMSO on current. (a) Ikr currents of WT was recorded when extracellular solution contained 0 (total solution volume: 3000 µl) and 25.0 µl DMSO (total solution volume: 3025 µl, 0.82% v/v) respectively. (b) It was not found that there was significant change of current amplitude under the two conditions ($n = 5$, $p > 0.05$).
